# Threonine appears to be essential for proliferation of human as well as mouse embryonic stem cells

**DOI:** 10.3389/fcell.2014.00018

**Published:** 2014-05-20

**Authors:** Lon J. Van Winkle, Vasil Galat, Philip M. Iannaccone

**Affiliations:** ^1^Department of Biochemistry, Midwestern UniversityDowners Grove, IL, USA; ^2^Developmental Biology Program, Department of Pathology, Lurie Children's Research Center, Feinberg School of Medicine, Northwestern UniversityChicago, IL, USA; ^3^Developmental Biology Program, Department of Pediatrics, Lurie Children's Research Center, Feinberg School of Medicine, Northwestern UniversityChicago, IL, USA

**Keywords:** threonine, embryonic stem cells, cell proliferation, humans, amino acid transport systems

## Introduction

Mouse embryonic stem (mES) cell proliferation depends exclusively on the nutritionally essential amino acid, L-threonine, in the medium. Other essential and non-essential amino acids need not be added to the medium for mES cell proliferation. Furthermore, the threonine analog, 3-hydroxynorvaline (3-HNV), selectively inhibits mES cell proliferation (Wang et al., 2009). HeLa, MEF and 3T3 cell growth all are not affected by 3-HNV. Selective inhibition of ES cell proliferation by 3-HNV is expected to have teratogenic and embryotoxic effects on development, and these effects have been observed in chicken and mouse embryos (Louw et al., 2005).

It has been proposed that rapid catabolism via threonine dehydrogenase (TDH) accounts for how threonine supplementation is needed to support mES cell proliferation and that TDH is the site of 3-HNV inhibition of mES cell proliferation (Wang et al., 2009; Han et al., 2013; Chen and Wang, 2014). In support of this possibility, TDH induction enhances reprogramming of mouse somatic cells into induced pluripotent stem (iPS) cells, and 3-HNV inhibits this induction (Han et al., 2013). The concentrations of threonine or 3-HNV needed for mES cell proliferation or its inhibition are, however, over an order of magnitude lower than the apparent *K*_m_ or *K*_i_ values for interaction of threonine or 3-HNV with TDH (Wang et al., 2009). Hence, another mechanism may account for the influences of threonine and 3-HNV on mES cell proliferation. To test this hypothesis, we determined whether 3-HNV inhibits human embryonic stem (hES) cell proliferation. Humans produce truncated and apparently inactive TDH proteins that cannot make appropriate contact with 3-HNV, threonine or NAD^+^, the cofactor needed for TDH activity (Edgar, 2002).

## Materials and methods

**Figure 1:**
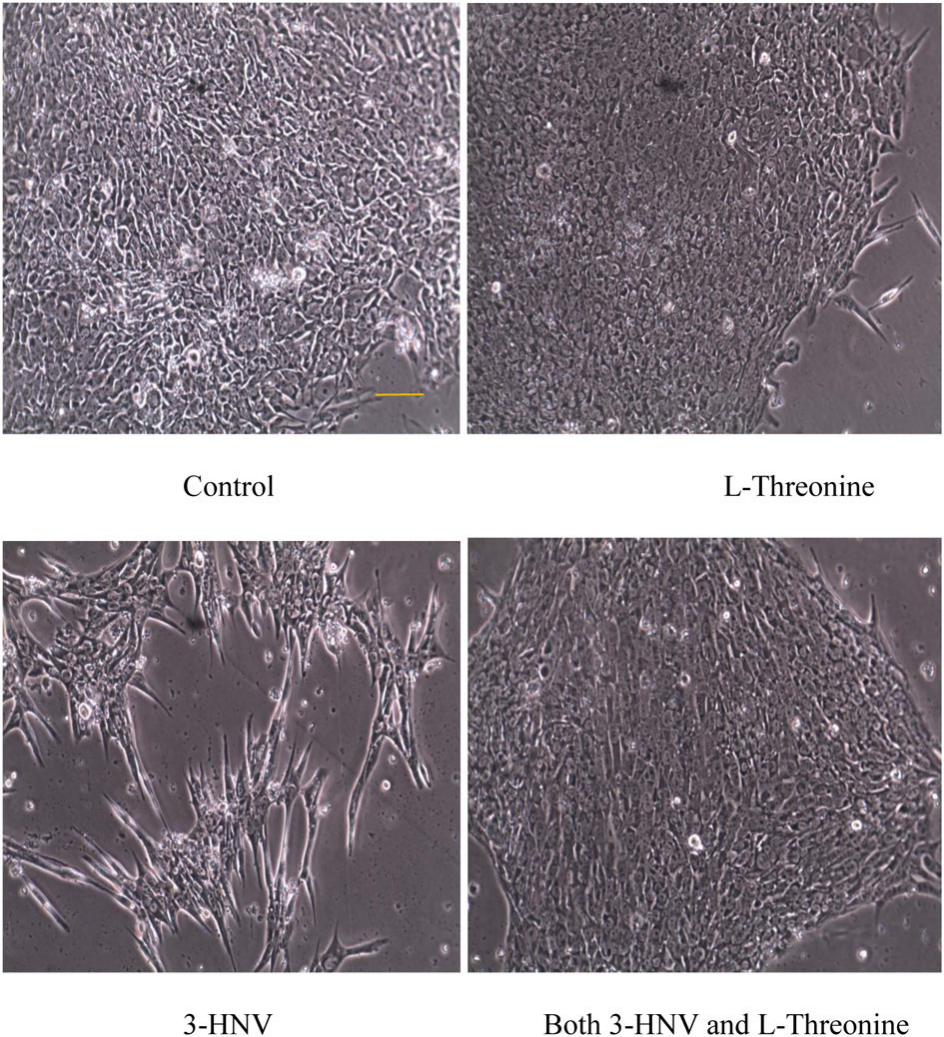
**hES cell and colony morphology after 2 days of culture in complete Stem-Pro medium (control) or this medium containing 4.0 mM L-threonine, 3-HNV or both 3-HNV and L-threonine**. Threonine (4.0 mM) rescued hES cells from 4.0 mM 3-HNV. (Scale bar = 50 um)

hES cells (H9 cell line, WA09) were maintained in complete Stem-Pro medium, DMEM/F12, supplemented with GlutaMax,™ Stem Pro^®^Supplement, Bovine Serum Albumin, FGF, and 2,β-Mercapthneol; the medium was changed daily. Human ES cells were cultured for 3 days prior to removal of medium and addition of experimental culture media. ES cells were cultured without mouse feeder cells. The diameters of all colonies were measured daily for 3 days using an inverted microscope. Each treatment group included at least six colonies in each of three independent experiments (total of at least 18 colonies in each group). The mean colony diameters of each of the four groups indicated in Figure 1 were compared statistically using analysis of variance (except the 3-HNV treatment group on day 3).

## Results and discussion

The diameters of all colonies were measured daily for 3 days using an inverted microscope (except the 3-HNV treatment group on day 3). Four mM 3-HNV inhibited growth of hES cell colonies over the first 2 days of treatment (*p* < 0.0001). Moreover, hES cell colonies lost integrity and dissociated after 3 days of treatment with 4.0 mM 3-HNV. All such colonies appeared to be dead. Four mM L-threonine largely rescued hES cells from 3-HNV inhibition (*p* < 0.0001) and apparent toxicity. This threonine rescue from 3-HNV included elongation and apparent differentiation of a greater proportion of rescued hES cells relative to control hES cells, so the rescue was likely incomplete (e.g., Figure 1). Nevertheless, TDH cannot be the site of 3-HNV action in hES cells nor can it account for the ability of threonine to rescue hES cells from 3-HNV. These observations open the question whether TDH is the only site of action of 3-HNV and threonine in mES cells. In this regard, threonine regulates the G1/S phase transition in mES cells in part through interaction with an amino acid transport system in the plasma membrane of the cells (Ryu and Han, 2011).

## Author contributions

Lon J. Van Winkle conceived and designed the study; collected and assembled the data with the help of Vasil Galat analyzed and interpreted the data with the help of Philip M. Iannaccone; and wrote the manuscript with the help of Philip M. Iannaccone and Vasil Galat; Vasil Galat: provided the stem cells and performed the experiments; Philip M. Iannaccone: provided financial and administrative support. All authors approved the manuscript in its final form and agree to be accountable for it.

### Conflict of interest statement

The authors declare that the research was conducted in the absence of any commercial or financial relationships that could be construed as a potential conflict of interest.
